# A new trick for an old lipid

**DOI:** 10.7554/eLife.22492

**Published:** 2016-11-25

**Authors:** Hayley Sharpe

**Affiliations:** Cambridge Institute for Medical Research, University of Cambridge, Cambridge, United Kingdomhjs49@cam.ac.uk

**Keywords:** cholesterol, G-protein coupled receptor, Hedgehog signaling, Human, Mouse

## Abstract

Cholesterol can regulate the Hedgehog signalling pathway by directly binding to a receptor on the cell surface.

**Related research article** Luchetti G, Sircar R, Kong JH, Nachtergaele S, Sagner A, Byrne EFX, Covey DF, Siebold C, Rohatgi R. 2016. Cholesterol activates the G-protein coupled receptor Smoothened to promote morphogenetic signaling. *eLife* **5**:e20304. doi: 10.7554/eLife.20304

Cholesterol is a lipid molecule that is a vital component of all animal cell membranes. It provides structural integrity, which is needed for the membrane to be an effective barrier, and is also required for the production of hormones and vitamin D. These roles mean the production and transport of cholesterol in cells is strictly regulated. This, combined with its poor solubility, has hindered efforts to study its specific molecular roles. Despite this, cholesterol has long been connected to the Hedgehog signalling pathway, which helps to regulate how tissues form in animals and is mutated in several types of cancer.

Now, in eLife, Rajat Rohatgi from Stanford University, Christian Siebold from the University of Oxford and colleagues – including Giovanni Luchetti and Ria Sircar as joint first authors – report a new role for cholesterol in activating the Hedgehog pathway through the receptor protein Smoothened (Luchetti et al., 2016). Similar results have also been recently reported by Adrian Salic and colleagues (Huang et al., 2016).

There are three main components in the Hedgehog pathway that allow cells to send and receive signals: the signalling protein Hedgehog, a transmembrane protein called Patched, and a transmembrane receptor protein called Smoothened. In the absence of Hedgehog, Patched inhibits Smoothened. However, when Hedgehog binds to Patched, this inhibition is blocked and Smoothened is able to activate other Hedgehog pathway components inside the cell. It is thought that Patched and Smoothened communicate using a small molecule rather than by direct contact (Taipale et al., 2002), but it is not clear exactly how this works.

Smoothened possesses two sites at which small molecules are able to bind: one is in its transmembrane domain region and the other is in its cysteine-rich domain on the external surface of the cell. A similar cysteine-rich domain is found in several other proteins, where it is known to be able to bind to lipids (Bazan et al., 2009). Earlier this year, Rohatgi, Siebold and colleagues presented the first complete crystal structure of the transmembrane domain region and cysteine-rich domain of Smoothened (Byrne et al., 2016). Unexpectedly, they found a cholesterol molecule occupied a hydrophobic (water-fearing) pocket in the cysteine-rich domain. Since disrupting cholesterol production in humans and mice affects Smoothened activity (Blassberg et al., 2016; Cooper et al., 2003), this raised the possibility that cholesterol might directly bind to and regulate Smoothened.

Cholesterol is a challenging molecule to work with because it is hydrophobic and can randomly integrate into membranes and modify the activities of many proteins. To overcome this problem both Luchetti et al. and Huang et al. used a chemical called methyl-β-cyclodextrin to deliver cholesterol to cells and show that it directly activates Smoothened through its cysteine-rich domain.

There are many common findings between the two studies. Firstly, both teams demonstrate that cholesterol stimulates Hedgehog signalling via Smoothened with a high degree of specificity. For example, cholestenol and other molecules that are similar to cholesterol were unable to do the same. Both teams were able to rule out the transmembrane domain region as the site of cholesterol binding by showing that cholesterol could activate Smoothened even in the presence of mutations that block the binding of small molecules to this region. By contrast, mutating or completely removing the cysteine-rich domain of Smoothened blocked both the cholesterol and Hedgehog responses. Furthermore, the presence of cholesterol and Hedgehog protein together led to higher levels of Hedgehog signalling activity than the presence of just Hedgehog protein, indicating a possible role for Patched in the regulation of Smoothened by cholesterol (Figure 1).

**Figure 1.**
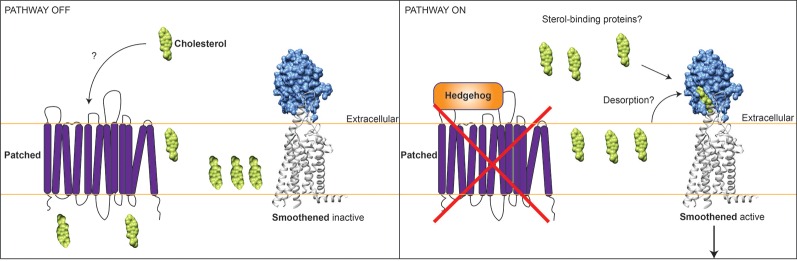
Model for how cholesterol may regulate the Hedgehog signalling pathway. Left: In the absence of Hedgehog protein, the transmembrane protein Patched (purple) inhibits the transmembrane receptor protein Smoothened (blue and grey) via an unknown mechanism. The findings of Luchetti et al. and Huang et al. suggest that Patched and Smoothened may communicate using the lipid molecule cholesterol (green), which is a core component of animal cell membranes (orange lines). Patched is similar to other proteins that transport small molecules across membranes, and might act to limit cholesterol access to Smoothened. In the absence of available cholesterol, Smoothened receptors on the cell surface are inactive. Right: Hedgehog protein binds to and inactivates Patched, potentially increasing cholesterol levels outside the cell. Cholesterol binds to a hydrophobic pocket in the Smoothened cysteine-rich domain (blue). Smoothened can now activate the Hedgehog signalling pathway, although many details of this process are not fully understood (see main article). In particular, it is not clear how cholesterol gains access to the Smoothened cysteine-rich domain. Cholesterol can be released from the membrane into the extracellular space by a process called desorption, but its insolubility makes this energetically unfavourable. Alternatively, a sterol-binding protein outside cells could deliver cholesterol to Smoothened.

How does cholesterol binding outside the cell translate to signalling within the cell? Luchetti et al. predict, based on previous structures (Byrne et al., 2016), that cholesterol binding to the cysteine-rich domain of Smoothened induces a clockwise rotation with respect to the transmembrane domain region. This change in shape could be sufficient to promote signalling inside the cell.

Together the findings of Luchetti et al. and Huang et al. strongly support a role for cholesterol in activating Smoothened in cells. However, it is worth noting that recent findings from other research groups favour an inhibitory role for sterol molecules instead (Roberts et al., 2016; Sever et al., 2016). Therefore, several critical questions remain. Does cholesterol binding itself alter Smoothened activity, or is cholesterol merely a cofactor that is needed for Smoothened to be activated by another molecule? Does Hedgehog protein affect cholesterol levels and is this mediated through the activity of Patched (Figure 1)? Since most cholesterol is trapped within the cell membrane, it will also be important to understand how cholesterol is able to access the cysteine-rich domain of Smoothened.

Nonetheless, this work reveals a new signalling role for cholesterol in controlling the Smoothened receptor and reiterates the possibility that Hedgehog signalling may have evolved from an ancient lipid-sensing pathway (Hausmann et al., 2009).
